# Aptamer-Based Delivery of Genes and Drugs Across the Blood–Brain Barrier

**DOI:** 10.3390/ph19010164

**Published:** 2026-01-16

**Authors:** Luona Yang, Yuan Yin, Xinli Liu, Bin Guo

**Affiliations:** Department of Pharmacological and Pharmaceutical Sciences, University of Houston, Houston, TX 77204, USA

**Keywords:** blood-brain barrier, aptamers, gene therapy, drug delivery, nanotechnology

## Abstract

The blood–brain barrier (BBB) restricts therapeutic delivery to the central nervous system (CNS), hindering the treatment of neurological disorders, such as Alzheimer’s disease, Parkinson’s disease, brain cancers, and stroke. Aptamers, short single-stranded DNA or RNA oligonucleotides that can fold into unique 3D shapes and bind to specific target molecules, offer high affinity and specificity, low immunogenicity, and promising BBB penetration via receptor-mediated transcytosis targeting receptors such as the transferrin receptor (TfR) and low-density lipoprotein receptor-related protein 1 (LRP1). This review examines aptamer design through the Systematic Evolution of Ligands by Exponential Enrichment (SELEX) and its variants, mechanisms of BBB crossing, and applications in CNS disorders. Recent advances, including in silico optimization, in vivo SELEX, BBB chip-based MPS-SELEX, and nanoparticle–aptamer hybrids, have identified brain-penetrating aptamers and enhanced the brain delivery efficiency. This review highlights the potential of aptamers to transform CNS-targeted therapies.

## 1. Introduction

**Figure 1:**
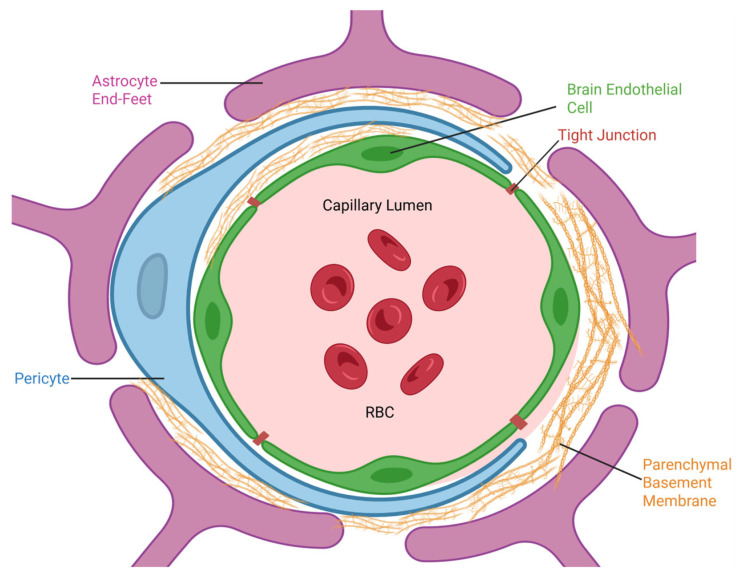
Structure of the blood–brain barrier (BBB), depicting the BBB components: endothelial cells with tight junctions, astrocytes, pericytes, and the parenchymal basement membrane.

The blood–brain barrier (BBB) is a selective interface that regulates molecular transport between the bloodstream and the central nervous system (CNS), protecting the brain from harmful substances. Composed of endothelial cells with tight junctions (e.g., claudin-5, occludin), astrocytes, pericytes, and a basement membrane (Figure 1), the BBB lacks fenestrations and restricts over 98% of small-molecule drugs and nearly all large-molecule therapeutics, such as antibodies and gene therapies [1,2]. This barrier, while essential for CNS homeostasis, severely limits the effective treatment of neurological disorders, which represent the leading global cause of disability and the second-leading cause of death. Conditions such as Alzheimer’s disease, Parkinson’s disease, brain tumors, stroke, and other neuropsychiatric disorders affect hundreds of millions worldwide, imposing enormous clinical urgency and socioeconomic burden.

The BBB poses a major bottleneck in CNS drug development, contributing to exceptionally high clinical failure rates—approximately 85–100% for many indications in Phase II/III trials—and prolonged development timelines compared to non-CNS therapeutics. Consequently, few effective treatments exist for most CNS disorders, underscoring the need for innovative, targeted delivery strategies capable of achieving therapeutic brain concentrations without compromising barrier integrity or causing systemic toxicity.

Traditional CNS delivery methods such as liposomes, polymeric nanoparticles, and viral vectors face significant challenges. Liposomes lack specificity and showed limited brain penetration [3]. Polymeric nanoparticles offer design flexibility but exhibit inconsistent BBB crossing and potential toxicity [4]. Viral vectors, such as adeno-associated viruses, enable efficient CNS gene delivery, but raise concerns about immunogenicity and limited cargo capacity [5]. These limitations underscore the need for targeted, low-toxicity CNS delivery systems (Table 1).

**Figure 2:**
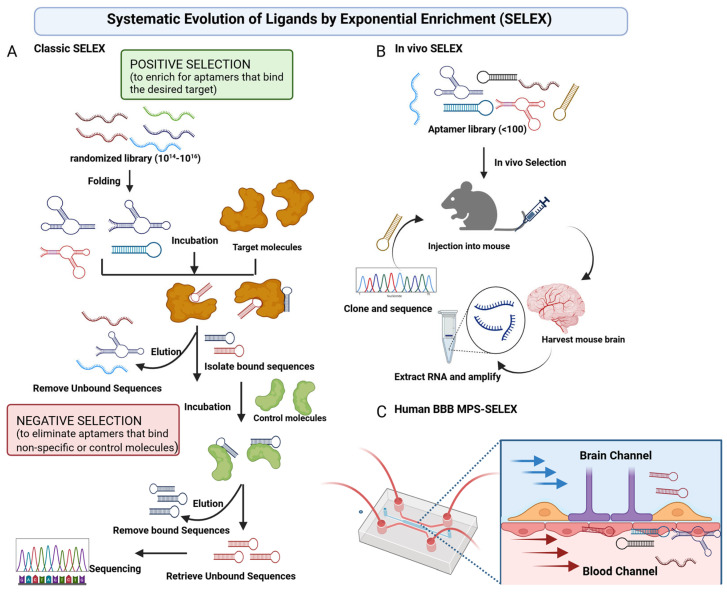
(**A**) Schematic representation of the classic Systematic Evolution of Ligands by Exponential Enrichment (SELEX) process, showing steps including library incubation with target, binding, partitioning of bound aptamers, and PCR amplification. (**B**) Schematic representation of in vivo SELEX for identification of brain-penetrating aptamers in mice. (**C**) Schematic diagram of microphysiological system-based SELEX (MPS-SELEX) process for identification of brain shuttle aptamers using human BBB chip.

Aptamers, single-stranded DNA or RNA oligonucleotides (20–100 nucleotides), bind specific molecular targets with high affinity (picomolar to nanomolar range) and low immunogenicity [6]. The name aptamer derives from the Latin word aptus (to fit) and the Greek word meros (part). The target binding affinity and specificity of aptamer relies on its unique capability to fold into a stable three-dimensional structure such as hairpins, stem-loops, G-quadruplex, pseudoknots, bulges, three-way junction (3WJ), etc., this unique sequence-dependent 3D folding allows aptamer to fit precisely with their target molecule binding pockets, bind to non-nucleotide target molecules with high affinity and specificity [9,15]. The folding is driven by the formation of complementary base pairs organized into secondary structure [16,17]. Although aptamers (~5–20 kDa) are commonly termed “chemical antibodies” or “nucleic acid antibodies” due to their unique binding and selectivity to different target molecules including proteins, small molecules, carbohydrates, or whole cells, they offer distinct advantages over antibodies (~150 kDa) in terms of ease of synthesis and modification, low cost, robustness, and easier entry into biological compartments. Aptamers are typically discovered from vast random nucleic acid sequence libraries using an iterative in vitro selection process called Selected via Systematic Evolution of Ligands by Exponential Enrichment (SELEX) (Figure 2A) [18]. Aptamers are versatile molecules with different applications spanning therapeutics as agonists/antagonists, targeted drug delivery, diagnostics, biosensors, and basic research. As therapeutics, FDA has approved two intravitreally injected RNA aptamers including VEGF-targeted pegaptanib for neovascular age-related macular degeneration (AMD) in 2004 and avacincaptad pegol to inhibit complement protein C5 for geographic atrophy secondary to AMD in 2023 [19,20]. Unlike some protein therapeutics, the immunogenicity of chemically modified DNA/RNA aptamers is relatively low, and the immune system generally does not mount strong B- or T-cell response against short oligonucleotides, which lack foreign protein epitopes [21]. Innate immune activation occurs due to the activation of Toll-like receptors, aptamers generally contain sugars modified at their 2′-position of the ribose sugar, the modification and exclusion of CpG motifs sometimes abrogate the TLR response [6]. As drug delivery carriers, aptamers were found to have potential to shuttle therapeutics across the BBB by targeting endogenous BBB receptors, such as transferrin receptor (TfR) and low-density lipoprotein receptor-related protein 1 (LRP1), facilitating receptor-mediated transcytosis [9]. This review examines aptamer design, BBB-crossing mechanisms, applications in neurological disorders, recent advancements, and challenges, highlighting their potential for effective CNS-targeted drug and gene delivery.

## 2. Aptamer Design and Selection

### 2.1. SELEX and Its Variants

The classic SELEX (Figure 2A), introduced in the 1990s, is an in vitro selection technique for isolating high-affinity nucleic acid aptamers from a randomized oligonucleotide library (10^14^–10^16^ sequences, 20–100 nucleotides) [22]. The process involves incubating the library with a target (e.g., purified protein such as a receptor), partitioning the bound aptamers using filtration, magnetic beads, or electrophoresis, and amplifying the bound fraction via polymerase chain reaction. Iterative cycles (8–15) with increasing stringency enrich aptamers with dissociation constants, often in the nanomolar to picomolar range [23]. High-throughput sequencing and bioinformatics were used to refine the pool and identify sequences with optimal target affinity. Classical SELEX, while effective for purified targets, lacks the physiological context for complex systems such as the BBB. Variants like cell-SELEX address this issue by using whole cells, such as human brain microvascular endothelial cells (hBMECs) hCMEC/D3, and mouse brain microvanscular endothelial cell bEND3 to select endothelial cell-internalizing aptamers such as R11-3 [24]. The living cells allow investigators to identify receptor-specific aptamers that bind to the target protein in its native active conformation in a specific cell type of interest. The whole-cell-based SELEX can be applied to uncharacterized target proteins as the selection process relies on the differences between the target cell population and a negative cell population.

### 2.2. In Vivo SELEX and Microfluidic-Based Aptamer Selection

In vivo SELEX screening approaches, conducted in animal models, have also been reported to select the brain-targeting aptamers (Figure 2B). Cheng et al. developed a library of aptamers and an in vivo evolution protocol to determine whether specific brain homing aptamers could be identified after injection into the peripheral vasculature. The library consists of RNA aptamer sequences with 2′-fluoro nucleosides to enhance nuclease resistance. Purified RNAs were peripherally injected into C57BL/6 mice and circulated for 1–3 h; then, mice were perfused with buffer and the RNAs were extracted from whole brain, the recovered RNAs were treated with RNAse A and DNase I and then converted to double-stranded DNA by RT-PCR; purified DNAs were transcribed into 2′-fluoro RNA; and full-length RNA was purified for the subsequent round of biopanning. After more than 15 rounds of in vivo selection, multiple aptamers were identified to bound to brain capillary endothelia and penetrated into the parenchyma [25]. In vivo SELEX accounts for systemic circulation and BBB interactions, isolating aptamers against receptors such as TfR or LRP1 with enhanced brain penetration [9,25].

Innovations such as microfluidic-based SELEX and toggle-SELEX enable high-throughput screening and dual-target specificity, respectively, enhancing aptamer development for CNS delivery [26]. The microfluidic-based, physiologically relevant, and novel MPS-SELEX approach was reported to identify BBB penetrating aptamers (Figure 2C). A human MPS-SELEX system was built on a poly(dimethylsiloxane) (PDMS) microfluidic chip; this microphysiological system (MPS) technology that lined with induced pluripotent stem cell (iPSC)-derived human BMECs (iPSC-BMECs) interfaced with primary human brain astrocytes and pericytes can mimic the human BBB. The MPS-SELEX procedure uses single-stranded DNA (ssDNA) library or the PCR-amplified pool flowed into the blood channel of the BBB MPS, and the BBB permeable ssDNA is collected from brain channel efflux and amplified by PCR for next round of selection. The sequences of collected ssDNA after multiple rounds of SELEX are obtained using NGS. This MPS-SELEX successfully identified a BBB shuttle aptamer, hBS01, which binds to the BMECs and efficiently penetrates the brain and binds to human brain cells under physiological shear stress. The hBS01 aptamer facilitated clathrin-mediated transcytosis, achieving 10-fold enhanced protein cargo transport in vitro compared with non-specific aptamers [10]. The comparison of different SELEX platforms for brain-targeting aptamer selection is summarized in Table 2. These advancements have established SELEX as an essential tool for designing BBB-targeted aptamers.

### 2.3. DNA vs. RNA Aptamers

DNA and RNA aptamers, single-stranded oligonucleotides (20–100 nucleotides), achieve target recognition through sequence-specific folding but differ in properties critical for BBB delivery (Table 3). Both rely on Watson–Crick base pairing, yet their structural and stability profiles influence their suitability for CNS applications [27].

DNA aptamers, composed of deoxyribonucleotides, form stable secondary structures such as stem-loops and G-quadruplexes, offering high thermal stability (melting temperatures >60 °C) and binding affinities (K_d_ ~1–100 nM). Their lack of a 2′-hydroxyl group enhances resistance to serum nucleases, yielding half-lives of several hours in vivo without modifications [7]. Cost-effective synthesis (~USD 0.01 per nucleotide) makes DNA aptamers well-suited for sustained BBB shuttling, such as in chronic neurological disorders. For instance, a TfR-targeted G-quadruplex-proximized DNA aptamer (G4PA) expressed a high targeting capacity for TfR [30].

RNA aptamers, with ribose sugars, adopt complex tertiary structures (e.g., helices and non-canonical motifs), enabling versatile binding to diverse targets, including BBB receptors such as LRP1 [28]. However, their 2′-hydroxyl group renders them susceptible to RNase degradation, with half-lives of minutes unless otherwise modified [29]. Chemical modifications, such as 2′-fluoro or 2′-O-methyl substitutions, extend the half-lives significantly while retaining ~80–90% binding affinity [13]. Modified RNA aptamers targeting insulin receptors have enhanced BBB transcytosis in rodent models [9]. Chimeric DNA-RNA aptamers combine stability and versatility, enhancing their applicability in BBB delivery. The choice between DNA and RNA aptamers depends on the stability needs vs. binding complexity for CNS therapeutics.

### 2.4. Computational Approaches in Aptamer Design

Computational approaches are increasingly used to enhance aptamer design by enabling high-throughput virtual screening of vast sequence spaces (10^14^–10^16^ possibilities), optimizing specificity for targets such as BBB transporters TfR and LRP1. In silico workflows typically include sequence generation, structure prediction, docking to targets, and iterative optimization via mutations or modifications. These in silico approaches enable de novo aptamer design, refinement of sequences, prediction of folding, optimizing binding affinity and selectivity, and simulation of targeting interactions [34,35,36].

#### 2.4.1. Specific Algorithms and Tools

Machine learning (ML) and deep learning (DL) algorithms analyze SELEX and sequencing data to identify high-affinity aptamers with high accuracy in controlled settings. Traditional ML methods, such as Support Vector Machines (SVM) and Random Forests, classify binders vs. non-binders based on sequence features and structural descriptors. More advanced DL frameworks include convolutional neural networks (CNNs) for motif detection and bidirectional long short-term memory (BiLSTM) networks for sequential dependencies.

Notable tools include the following:

1. DeepAptamer: A hybrid CNN-BiLSTM model that integrates sequence composition and secondary structure features to predict binding affinities from early, unenriched SELEX rounds. It identifies high-affinity aptamers against diverse targets, potentially bypassing 20–30 iterative SELEX cycles needed for full enrichment [37].
2. RaptGen: A variational autoencoder (VAE) with a profile-hidden Markov model decoder that generates novel aptamer sequences from latent space representations of HT-SELEX data, capturing motifs and enabling Bayesian optimization for affinity-guided variants [38].
3. AptaSim: Simulates SELEX processes in silico, including error-prone PCR and mutation rates, to predict enriched pools and export candidate sequences for validation [39].
4. Other emerging models: AptaTrans (transformer-based pretrained encoders for aptamer–protein interaction prediction) [40], AptaDiff (diffusion-based de novo design) [41], and generative adversarial networks for modified nucleotides.

Complementing ML, molecular dynamics (MD) simulations provide structural insights into aptamer–target interactions. Simulations of RNA aptamer–receptor complexes have identified stabilizing hydrogen bonds, yielding variants with improved affinity [32,42]. Docking tools such as the accurate tool (e.g., molecular docking) predict aptamer–nanoparticle conjugate interactions, supporting TfR-targeted delivery with improved brain penetration in mouse models [43].

Aptamer virtual screening and de novo design are challenging due to the poor understanding of nucleic acid sequence/folding and structural-binding function. Deep learning models such as MXfold2, UFold, and SPOT-RNA have enhanced nucleic acid secondary structure prediction; 3D prediction models such as RoseTTAFoldNA and AlphaFold 3 have shown remarkable capabilities for predicting the 3D structures of nucleic acids [34,44,45,46,47]. Generative models, such as variational autoencoders and generative adversarial networks, design modified nucleotides (e.g., 2′-fluoro RNA) to enhance nuclease resistance for BBB applications [48].

#### 2.4.2. Practical Impact on Timelines and Efficacy

These computational tools dramatically accelerate aptamer discovery by reducing experimental SELEX cycles from 12 to 15 to as few as 4–7 in AI-assisted protocols, shortening timelines from months to weeks while lowering costs. In silico prediction enables the screening of millions of sequences virtually, identifying candidates with nanomolar to picomolar affinities and improved binding affinity (e.g., 1.5–5-fold lower K_d_) [34,35,36]. Efficacy gains include better specificity, reduced off-target effects, and enhanced stability, as validated in preclinical models for BBB shuttles. Integration with experimental methods like microfluidic-SELEX further amplifies success rates.

Challenges include limited training datasets, scarcity of single-stranded oligonucleotide structural data, and the need for physiological validation. Overall, integration of computational prediction power with novel experimental approaches such as microfluidic-SELEX will provide a powerful platform for advancing the discovery of novel aptamers and enhance their potential for CNS-targeted drug and gene delivery.

## 3. Mechanisms of Aptamer-Mediated BBB Crossing

Aptamers represent a versatile class of ligands that exploit the endogenous transport machinery of the BBB to deliver therapeutics to the CNS, circumventing the barrier’s restrictive nature without invasive interventions. The primary mechanism is receptor-mediated transcytosis (RMT), a saturable, energy-dependent process involving binding to luminal receptors on brain endothelial cells, clathrin- or caveolin-mediated endocytosis, vesicular trafficking across the cell (typically 10–30 min), and exocytosis into the brain interstitium [49]. This pathway achieves 0.5–5% brain uptake in preclinical models, far surpassing passive diffusion (<0.1% for most molecules). The small size (6–30 kDa) and high affinity (K_d_ 1–100 nM) of aptamers enable efficient receptor engagement while minimizing steric hindrance, with post-binding dissociation rates tuned to favor transcytosis over recycling. Secondary mechanisms, such as adsorptive transcytosis or paracellular modulation, complement RMT but could carry higher toxicity risks. Below, we outline the key receptors and conjugation strategies central to these processes.

### 3.1. Key Receptors for RMT

Aptamers are selected or engineered to mimic natural ligands for BBB-enriched receptors that mediate nutrient and waste transport. These targets such as TfR, LRP1, insulin receptor (IR), and glucose transporter 1 (GLUT1) differ in their expression levels (10^4^–10^6^ copies/cell), cargo preference, and regulatory cues, allowing context-specific selection.

Transferrin Receptor (TfR): As a prototypical RMT target, uniform high expression of TfR in the brain endothelium (~200,000 sites/cell) supports iron homeostasis and is hijacked for non-iron cargoes [50]. DNA aptamers against TfR bind the receptor with nanomolar affinity, inducing clathrin-coated pit formation and enhanced transcytotic flux of conjugates (e.g., ~80% in vitro for ProSNAs) [42]. In vivo, intravenous administration of TfR aptamer-functionalized protein spherical nucleic acids (ProSNAs) in CD1 mice resulted in significantly enhanced brain accumulation (5–7-fold over non-targeted controls), with confocal microscopy confirming widespread parenchymal distribution, perivascular escape, and neuronal uptake without altering TfR trafficking [42].

Low-Density Lipoprotein Receptor-related Protein 1 (LRP1): LRP1 is a multifunctional receptor highly expressed on brain endothelial cells and plays a key role in shuttling large cargoes via caveolar and clathrin-mediated endocytosis, making it suitable for aptamer–payload hybrids [43]. LRP1 is central to endogenous amyloid-β clearance from the brain across the BBB, with endothelial-specific LRP1 deletion in mice significantly impairing amyloid-β efflux and exacerbating brain accumulation [51]. This physiological role may confer certain advantages for LRP1-targeted compared to TfR aptamers, such as potentially higher capacity for larger cargoes due to differential vesicular trafficking. Preclinical studies with LRP1 ligands (e.g., Angiopep-2 peptides) have demonstrated effective brain penetration, supporting its promise for aptamer-based delivery [52].

#### Insulin Receptor (IR) and Glucose Transporter 1 (GLUT1)

The insulin receptor (IR) is expressed on brain endothelial cells and mediates saturable, receptor-mediated transcytosis of insulin, though the exact mechanism and contribution of the signaling-related IR remain debated [53,54]. IR expression and insulin transport can be modulated under conditions of metabolic stress, such as diabetes or hyperglycemia. Aptamers targeting the IR have been developed as agonists or allosteric modulators with high affinity, inducing biased signaling in cellular models [33]. However, their use for BBB transcytosis or cargo delivery has not been demonstrated. GLUT1, the primary facilitator of glucose transport across the BBB, is highly expressed in brain endothelium and supports basal glucose flux essential for CNS energy needs [55]. While glucose-derivatized nanoparticles exploit GLUT1 for enhanced BBB penetration, specific GLUT1-targeted aptamers for therapeutic delivery remain unreported.

### 3.2. Conjugation Strategies for Enhanced Payload Delivery

The limited intrinsic capacity of aptamers (~100–500 nt equivalents) is overcome by conjugation to nanocarriers, which shield payloads from degradation and amplify multivalency. Linkers such as PEG spacers or pH-cleavable hydrazones maintain high aptamer functionality while enabling triggered release [9]. For example, TfR aptamer-functionalized biomimetic nanocomplexes have delivered siRNA across the BBB in glioblastoma models, achieving gene silencing, apoptosis, and antitumor efficacy [56]. TfR aptamer-conjugated protein spherical nucleic acids (ProSNAs) have also demonstrated enhanced brain penetration and parenchymal distribution in vivo [42]. Receptor-targeted systems (e.g., LRP1 via ligands like Angiopep-2) using liposomes or nanoparticles show promise for CRISPR component delivery to the brain [9]. Amphiphilic aptamer-based conjugates improve solubility and targeting for hydrophobic drugs like paclitaxel in preclinical models.

### 3.3. Alternative Mechanisms: Paracellular Transport

Certain ligands or agents can modulate paracellular permeability by disrupting tight junction scaffolds (e.g., claudin-5, occludin, ZO-1), transiently increasing pore size and Papp (e.g., 3–5-fold in models) [46,57]. This can occur without proteases, via electrostatic or peptide interactions. However, in vivo translation is limited by risks like vasogenic edema; optimization (e.g., dose-titration, co-stabilizers) has enabled transient delivery of tracers like dextrans in preclinical stroke models, though neurotoxicity remains a concern [46].

Thus, aptamer-mediated mechanisms predominantly rely on targeted RMT, with paracellular approaches carrying higher toxicity risks and limited to non-aptamer agents. Hybrids remain poised for diverse CNS payloads, refined by multi-omics, PK/PD, and toxicology evaluation for clinical translation.

## 4. Applications in Gene Delivery

Aptamers have transformed targeted gene delivery to the CNS by exploiting their high specificity and ability to cross the BBB to addressing critical unmet needs in treating neurological disorders and brain cancers. Unlike viral vectors, which pose the risks of immunogenicity and insertional mutagenesis, aptamers provide a non-immunogenic, scalable platform with tunable pharmacokinetics for delivering nucleic acid therapeutics. DNA aptamers, known for their stability, are ideal for delivering large constructs such as plasmids and CRISPR/Cas9 systems, whereas RNA aptamers, with their structural versatility, excel in transporting smaller cargos including siRNA and miRNA. Despite their promise, challenges such as endosomal entrapment and transient gene silencing effects remain, necessitating innovative strategies to enhance delivery efficiency. This section explores the applications of DNA and RNA aptamers in CNS gene delivery and approaches to overcoming the current limitations.

### 4.1. DNA Aptamer-Based Gene Delivery

DNA aptamers are highly effective for CNS gene delivery due to their robust stability against nucleases and their ability to target BBB-specific receptors like TfR. For instance, microphysiological system-based SELEX was employed to identify DNA aptamers that facilitate transcytosis across the BBB, enabling efficient delivery of nucleic acid therapeutics to brain tissue [10]. Similarly, circular DNA aptamers targeting TfR was developed to deliver tau-disrupting genes in tauopathy mouse models, achieving enhanced BBB penetration and improved cognitive outcomes [31]. These aptamers have also been integrated into nanoplatforms, such as spherical nucleic acids (SNAs) to improve brain targeting of protein-coding genes [42]. Nafee et al. reviewed the potential of AS1411 DNA aptamers and aptamer-conjugated nanosystems for targeted delivery of therapeutics, including suicide genes (e.g., saporin toxin), to glioblastoma cells such as U87MG with high specificity and minimal off-target toxicity [12]. Additionally, Banik et al. employed exosome-encapsulated DNA aptamers to cross the BBB for ATP-sensing and potential siRNA delivery in Alzheimer’s disease models [32]. These studies underscore the potential and versatility of DNA aptamers for the stable, scalable delivery of large genetic constructs, although challenges like endosomal escape require further optimization, conjugation with fusogenic peptides or pH-sensitive nanocarriers could offer a mitigation strategy.

### 4.2. RNA Aptamer-Based Gene Delivery

RNA aptamers, characterized by their conformational flexibility and high-affinity binding, are particularly suited for delivering smaller nucleic acid cargos like siRNA and miRNA across the BBB. Ding et al. developed an RNA triple-helix hydrogel functionalized with RNA aptamers targeting CXCR4 for siRNA delivery in triple-negative breast cancer, demonstrating controlled gene silencing and metastasis inhibition [47], a strategy adaptable for CNS applications. Nafee et al. reviewed RNA aptamer-conjugated nanosystems, such as liposomes and nanoplexes, which leverage receptor-mediated transcytosis to deliver siRNA for neurodegenerative diseases and gliomas, emphasizing their role in non-viral gene therapy [12]. RNA aptamers offer advantages in binding to dynamic targets due to their structural adaptability, but their susceptibility to nuclease degradation necessitates chemical modifications such as 2′-fluoro, 2′-O-methyl, or phosphorothioate backbones. While RNA aptamers have shown promise in peripheral cancers, their application in CNS delivery is less developed compared to DNA aptamers, partly due to stability challenges in the harsh brain microenvironment. Innovations such as nanoparticle encapsulation and aptamer-hydrogel systems are critical for enhancing RNA aptamer stability and BBB penetration.

### 4.3. Challenges in Gene Delivery

Despite these advancements, several challenges have hindered the clinical translation of aptamer-based gene delivery. Endosomal entrapment remains a significant barrier, as aptamer-cargo complexes often become trapped in endosomes post-transcytosis, reducing therapeutic efficacy. Strategies such as the incorporation of endosomal-disrupting peptides or pH-responsive nanoparticles are being explored to address this issue [12]. Transient silencing effects, particularly with siRNA delivered by RNA aptamers, limit long-term therapeutic outcomes, necessitating repeated dosing or integration with stable expression systems like CRISPR/Cas9, where DNA aptamers excel [42]. Additionally, the heterogeneity of the BBB across disease states complicates aptamer design, as receptor expression (e.g., TfR) varies in conditions like Alzheimer’s or glioblastoma [32]. Scalability and cost of aptamer production, especially for RNA aptamers requiring extensive modifications, also pose challenges. Future directions include optimizing SELEX for BBB-specific aptamers [10] and developing hybrid DNA-RNA aptamer systems to combine stability and binding selectivity, ultimately enhancing the CNS gene delivery efficiency.

## 5. Applications in Drug Delivery

Aptamer-based platforms have been explored for the CNS delivery of small molecules, proteins, peptides, and antibodies. This section explores aptamer applications in delivering various therapeutic cargos across the BBB.

### 5.1. Small-Molecule Delivery

Aptamers can encapsulate or conjugate small-molecule drugs such as chemotherapeutics for targeted delivery and minimized systemic toxicity. For example, AS1411 DNA aptamer-conjugated nanosystems have been explored for delivering toxins or chemotherapeutic agents (e.g., mimicking saporin or paclitaxel action) to glioblastoma cells, achieving enhanced tumor targeting and reduced off-target effects [12]. Similarly, aptamer-functionalized nanoparticles have delivered hydrophobic drugs like camptothecin or paclitaxel in cancer models, with strategies leveraging receptor-mediated transcytosis potentially adaptable for brain tumors and metastases [12]. For instance, AS1411 aptamer-targeted rod-shaped mesoporous silica nanoparticles enabled co-delivery of camptothecin and surviving shRNA, resulting in synergistic antitumor effects, enhanced cellular uptake, and tumor suppression in colon adenocarcinoma models [58]. These systems ensure controlled drug release at the target site, although endosomal entrapment remains a hurdle, addressable via pH-sensitive carriers and innovative release strategies [12].

### 5.2. Protein and Peptide Delivery

Therapeutic proteins and peptides, such as brain-derived neurotrophic factor (BDNF) and anti-inflammatory peptides, are promising for neurological disorders but are limited by poor BBB penetration. DNA aptamers targeting LRP1 was used to deliver BDNF to neurons, promoting neuroprotection in stroke models [12]. Li developed circular DNA aptamers targeting TfR to deliver tau-disrupting cargos in tauopathy models, a platform adaptable for peptide delivery to modulate protein aggregation [31]. RNA aptamers excel with smaller cargos; Agrawal et al. described an RNA aptamer (anti-NgR)-loaded hyaluronic acid hydrogel for sustained peptide release in spinal cord injury models, achieving axonal regeneration via intrathecal delivery [59]. RNA aptamers have also delivered anti-inflammatory peptides in multiple sclerosis models, leveraging dual targeting and therapeutic functions [12]. Aptamer–protein conjugates often use cleavable linkers to ensure cargo activity, but RNA aptamer instability requires modifications like 2′-fluoro or 2′-methyl-modified backbones [12].

### 5.3. Antibody Delivery

Monoclonal antibodies targeting pathological proteins such as tau in Alzheimer’s disease are limited by poor BBB permeability. Aptamer–antibody conjugates combine aptamer specificity with antibody efficacy. Wang et al. utilized exosome-encapsulated DNA aptamers targeting TfR to cross the BBB in Alzheimer’s models, a strategy that is adaptable for anti-tau antibody delivery. Similarly, TfR-targeted bifunctional DNA aptamers have been developed to disrupt tau pathology in mice, highlighting potential platforms for antibody conjugation [31]. Randhir et al. showed that TfR-targeted DNA aptamers enhance BBB crossing of spherical nucleic acids, which is applicable to antibody cargos [42]. Choi et al. identified DNA aptamers via MPS-SELEX, optimizing antibody delivery to CNS tissues [10]. These hybrid systems mitigate macromolecule CNS penetration, but endosomal entrapment requires endosomal release solutions such as incorporation of fusogenic peptides [12].

## 6. Advantages and Challenges of Aptamer-Based Delivery Systems

Aptamer-based delivery systems provide key benefits for CNS therapeutics, particularly in overcoming the BBB. These advantages, rooted in their biochemical and biophysical properties, enhance their suitability for targeted drug and gene delivery. However, several challenges limit their clinical application, and addressing these hurdles is critical for advancing their therapeutic potential.

### 6.1. Advantages

1. High Specificity and Affinity: Aptamers bind to BBB receptors with dissociation constants often in the nanomolar to picomolar range, reducing non-specific binding to peripheral tissues and enhancing receptor-mediated transcytosis efficiency [10].
2. Low Immunogenicity: Unlike antibodies, aptamers elicit minimal immune responses owing to small size and chemical modification, enabling safe repeated dosing in chronic neurological conditions without significant immune-mediated clearance [12].
3. Chemical Modifiability: Aptamers can be modified with polyethylene glycol (PEG), cholesterol, or fluorophores to improve serum stability, extend circulation half-lives (e.g., >48 h for 2′-modified RNA), enable CNS imaging, and maintain functionality for BBB applications [14].
4. Cost-Effective Production: Scalable chemical synthesis of aptamers, costing ~USD 0.01 per nucleotide at scale, reduces expenses compared to recombinant antibody production, facilitating the large-scale development of CNS therapeutics [12].
5. Versatility: Aptamers support targeted delivery, diagnostic imaging, and theranostic applications in neurological disorders, such as glioblastoma or Alzheimer’s, by integrating receptor-specific targeting with payload delivery or metabolite sensing [32].

### 6.2. Challenges and Limitations

1. Nuclease Degradation: RNA aptamers are susceptible to degradation by serum and CNS nucleases, necessitating chemical modifications such as 2′-fluoro, 2′-methyl, or phosphorothioate backbones. These modifications, while extending half-life to >48 h, may slightly reduce binding affinity, impacting receptor-mediated transcytosis efficiency [12].
2. Limited Clinical Translation: Although aptamers such as pegaptanib and avacincaptad pegol are FDA-approved for non-CNS indications, few BBB-targeted aptamers have progressed to clinical trials due to challenges in stability, in vivo efficacy, good manufacturing practice (GMP)-compliant synthesis, and poor pharmacokinetics CNS applications [14]. Preclinical advances, such as TfR-targeted aptamers, are ongoing but require further validation.
3. BBB Heterogeneity: Variations in BBB receptor expression (e.g., TfR and LRP1) across species, disease states (e.g., glioblastoma vs. Alzheimer’s), and brain regions (e.g., cortex vs. hippocampus) complicate aptamer design and optimization, reducing consistency in transcytosis efficiency [32].
4. Payload Capacity: Aptamers have limited capacity (~100–500 nt equivalents) for large payloads like CRISPR complexes, requiring conjugation to nanoparticles to enhance delivery across the BBB, which may introduce complexity in formulation [42].
5. Pharmacokinetics: Unmodified aptamers undergo rapid renal clearance, reducing their circulation time to minutes. PEGylation or nanoparticle conjugation extends half-life but may alter biodistribution, necessitating careful optimization for CNS targeting [14]. Both FDA-approved aptamers pegaptanib and avacincaptad pegol are PEGylated oligonucleotides that bind to and inhibit their targets; PEGylation strategy could potentially improve the pharmacokinetics of promising aptamer-based CNS therapeutics.

## 7. Recent Advances and Future Directions

### 7.1. Aptamer–Nanoparticle Hybrids

Aptamer–nanoparticle hybrids have emerged as a promising approach for targeted delivery across the BBB, combining the high specificity of aptamers with the enhanced payload capacity and stability of nanoparticles to address the limitations of standalone aptamers, such as rapid renal clearance and restricted cargo volume. These systems leverage nanoparticle cores—such as gold nanoparticles (AuNPs), lipid nanoparticles (LNPs), or mesoporous silica nanoparticles functionalized with DNA or RNA aptamers targeting BBB receptors. Recent advances have demonstrated their potential for CNS drug and gene delivery, achieving improved brain penetration, reduced immunogenicity, and synergistic therapeutic effects in preclinical models of neurodegenerative diseases and brain tumors.

Randhir et al. demonstrated TfR-targeted DNA aptamers conjugated to protein spherical nucleic acids (SNAs), resulting in a 5–7-fold increased BBB penetration in vivo and sustained tau gene silencing in tauopathy mice, addressing endosomal escape through nanoparticle geometry optimization [42]. In lipid-based hybrids, Wang et al. engineered exosome-mimicking LNPs with TfR-specific DNA aptamers, delivering ATP-sensing probes and siRNA across the BBB and reducing amyloid-beta aggregates by 80% in AD mouse brains with minimal off-target effects [32]. Ray et al. developed RNA aptamer–peptide hybrids on LNPs targeting CCR5, a receptor overexpressed in neuroinflammation, achieving over 90% uptake in microglia for RNAi delivery in HIV-associated neurocognitive disorder models [11]. Inorganic nanoparticle hybrids also have advanced CNS delivery. Song et al. utilized circular DNA aptamers on AuNPs to co-deliver tau-disrupting peptides, improving cognitive outcomes by 40% in tauopathy models via TfR-mediated transcytosis and pH-responsive release [31]. Abbasi et al. reviewed aptamer–AuNP conjugates, noting 3–5-fold enhanced intracellular penetration via receptor-mediated endocytosis, a mechanism adaptable for CNS pathogens like Toxoplasma gondii [8].

Future research directions for aptamer–nanoparticle hybrids should focus on clinical scalability and multifunctionality. Integrating stimuli-responsive elements, such as enzyme-cleavable linkers or magnetic nanoparticles, can enable real-time theragnostic in dynamic CNS environments [60]. Machine learning-driven SELEX optimization will accelerate the design of patient-specific hybrids tailored to BBB heterogeneity in diseases like Parkinson’s and multiple sclerosis [10]. Combining aptamers with CRISPR/Cas9-loaded LNPs holds promise for gene editing across the BBB, but challenges like long-term biocompatibility and immune evasion require large-animal studies and GMP-scale production [61].

### 7.2. Computational and High-Throughput Approaches

Computational and high-throughput approaches have revolutionized aptamer development for CNS drug and gene delivery by enhancing the efficiency, specificity, and scalability of SELEX processes. These methods address the challenges of designing aptamers that cross the BBB with a high affinity for receptors like TfR, while overcoming limitations such as off-target binding and BBB heterogeneity across disease states. By integrating AI/ML, molecular dynamics simulations, and next-generation sequencing (NGS), these approaches enable rapid identification of high-affinity aptamers, reducing development time from months to weeks and improving binding kinetics by up to many folds significantly [34,35,36]. This section explores recent advances in computational and high-throughput strategies and their potential for personalized CNS therapeutics.

A key advancement is the use of MPS-SELEX coupled with computational modeling to identify DNA aptamers optimized for BBB transcytosis. Machine learning algorithms further refined aptamer selection by predicting secondary structures and binding kinetics, reducing the candidate pool by 80% while prioritizing sequences with dissociation constants below 10 nM [10]. High-throughput NGS and bioinformatics analysis of SELEX libraries further enable the detection of rare, high-affinity sequences that traditional methods may miss [34,35,36].

Molecular dynamics simulations and docking tools have accelerated aptamer optimization by predicting receptor interactions and guiding design for enhanced stability and affinity [34,35,36]. For example, computational approaches support TfR-targeted aptamer designs in spherical nucleic acid (SNA) and exosome-mimicking platforms, achieving improved BBB penetration and parenchymal distribution in preclinical models [32,42]. Computational modeling also addresses aptamer stability; for instance, chemical modifications like 2′-fluoro or 2′-O-methyl backbones can be optimized to significantly extend RNA half-life while preserving binding affinity [14]. These advances collectively reduce SELEX cycles from 12–15 to as few as 4–6 in AI-assisted protocols, lowering costs and enabling the rapid prototyping of BBB-specific aptamers [34,35,36].

Future research should focus on integrating computational and high-throughput approaches to overcome major challenges and limitations. First, advanced ML models, such as deep learning networks, can predict aptamer interactions with dynamic BBB receptors across CNS disease states such as Parkinson’s or glioma, accounting for receptor expression variability [34,35,36]. Second, combining single-cell RNA sequencing with NGS-SELEX can map BBB heterogeneity at the cellular level, enabling patient-specific aptamer design [1]. Third, computational platforms should prioritize aptamers for novel targets like occludin or GLUT1, potentially increasing BBB penetration efficiency by 2–3-fold. Fourth, high-throughput automation of aptamer–nanoparticle conjugation could scale production for clinical trials, building on the safety profile of approved aptamers like pegaptanib [14]. Finally, integrating computational aptamer design with CRISPR/Cas9 delivery systems holds promise for gene editing in CNS disorders, with preclinical studies showing 60% editing efficiency in neuronal cells [61].

### 7.3. Theragnostic Applications

Aptamers enable theragnostic applications by combining therapy and diagnostics in a single platform, allowing simultaneous real-time monitoring of drug delivery, therapeutic response, and disease progression. This integrated approach supports personalized medicine, particularly in challenging CNS disorders where precise targeting across the BBB is essential.

A prominent example is the TfR-targeted DNA aptamer developed by Banik et al., conjugated to fluorescent probes for enhanced brain penetration and visualization in preclinical models, demonstrating potential for simultaneous imaging and targeted payload release [32].

Beyond this, aptamer-functionalized nanoparticles have advanced theragnostic strategies for brain tumors. For instance, AS1411 aptamer-conjugated systems and similar hybrids have been explored for loading therapeutics (e.g., doxorubicin or paclitaxel) alongside imaging capabilities, showing improved tumor accumulation and controlled release in glioblastoma models [12]. TfR-aptamer or related hybrids with lipid carriers also facilitate concurrent diagnostic imaging and drug/gene delivery, achieving sustained parenchymal exposure in preclinical neuro-oncology studies [11].

These systems control aptamers’ high specificity to minimize systemic toxicity while providing feedback on treatment efficacy through integrated modalities. Ongoing refinements, such as stimuli-responsive linkers for triggered release and multimodal probes, hold promise for clinical translation in glioblastoma, Alzheimer’s disease, and other CNS pathologies, ultimately enabling adaptive, patient-specific therapies.

### 7.4. Translational Challenges and Clinical Progress

While aptamer-based BBB delivery has shown robust efficacy in preclinical models (e.g., enhanced brain penetration and therapeutic effects in rodent models of glioblastoma, tauopathy, and stroke), translational hurdles remain significant. Key challenges include interspecies differences in BBB receptor expression (e.g., TfR and LRP1), which complicate extrapolation from rodents to humans; potential saturation of receptor-mediated transcytosis at therapeutic doses; off-target accumulation in peripheral tissues with high receptor expression (e.g., liver for LRP1); and the need for rigorous evaluation of long-term immunogenicity and toxicity in higher species. Additionally, endosomal entrapment post-transcytosis and efficient intracellular cargo release continue to limit payload efficacy.

Despite these obstacles, early clinical progress is emerging for aptamers in neurological indications. The TLR4-antagonist aptamer ApTOLL, designed to reduce neuroinflammation and readily cross the BBB, has demonstrated neuroprotective effects in preclinical stroke models. A Phase I first-in-human study confirmed favorable pharmacokinetics and safety in healthy volunteers, and the completed Phase Ib/IIa APRIL trial showed positive safety and efficacy (e.g., reduced mortality and ifarct volume) as an adjunct to thrombectomy in acute ischemic stroke patients. The program was acquired by MERCK in 2024, with Phase IIb/3 trials anticipated. This represents the most advanced aptamer-based therapy targeting CNS pathology via BBB penetration. No TfR- or LRP1-targeted aptamer shuttles for drug/gene delivery have entered clinical trials for neurological disorders yet, underscoring the gap between promising preclinical data and human application. Addressing these translational barriers through humanized models, advanced MPS-SELEX and nanoparticle hybrids will be crucial for realizing aptamers’ clinical potential in CNS therapeutics.

## 8. Conclusions and Future Perspectives

Aptamer-based delivery systems represent a promising frontier for overcoming the BBB in CNS-targeted therapeutics. DNA aptamers offer stability and scalability, whereas RNA aptamers provide structural versatility and targeting precision. By exploiting receptor-mediated transcytosis and advanced conjugation strategies, these systems enable the efficient delivery of genes, small-molecule drugs, proteins, peptides, and antibodies to treat neurological disorders such as Alzheimer’s disease, Parkinson’s disease, glioblastoma, and stroke.

Despite significant preclinical progress, clinical translation remains limited by challenges including nuclease degradation (particularly for RNA aptamers), BBB heterogeneity across species and disease states, restricted payload capacity for large therapeutics, suboptimal pharmacokinetics due to rapid renal clearance, and the need for robust GMP-compliant manufacturing. Ongoing innovations—such as chemical modifications, PEGylation, nanoparticle hybrids, computational design, and high-throughput SELEX variants—are actively addressing these hurdles and enhancing brain penetration, stability, and therapeutic efficacy.

Looking ahead, several key directions will accelerate the development of aptamer-based CNS therapeutics:

1. Identification of aptamers for novel BBB targets

Including underexplored receptors (e.g., insulin receptor, GLUT1, occludin, claudin-5) and disease-specific epitopes to further enhance penetration and selectivity [10,33,61].

2. Optimization of aptamer physicochemical properties and pharmacokinetics

Minimizing nuclease degradation and renal clearance through advanced modifications (e.g., 2′-fluoro, 2′-O-methyl, PEGylation, cholesterol conjugation) and extended circulation strategies [7,13,14].

3. Enhancement of aptamer potency

Improving target accessibility, intracellular trafficking, folding optimization, and reduction in off-target binding via structure-guided design and affinity maturation [34,35,36,50].

4. Overcoming endosomal entrapment and enabling efficient intracellular cargo release

Incorporation of fusogenic peptides, pH-responsive linkers, or endosomal-disrupting agents to maximize therapeutic efficacy [11,12,54].

5. Improvement in scalability, rigor, and robustness of chemistry, manufacturing, and controls (CMC)

Establishing standardized GMP-compliant production processes to support clinical translation [14].

Emerging trends that are poised to transform the field include the following:

1. Aptamer–nanoparticle hybrids

Combining aptamers with lipid nanoparticles, spherical nucleic acids, or exosomes has demonstrated 5–10-fold improvements in brain delivery and payload capacity in preclinical models of Alzheimer’s disease, tauopathies, and glioblastoma [11,31,32]. These multifunctional platforms enable co-delivery of therapeutics and imaging agents, paving the way for personalized CNS treatments.

2. Machine learning-driven aptamer design

Deep learning models (e.g., DeepAptamer, generative models) trained on large SELEX datasets can now predict high-affinity aptamers with nanomolar binding in silico, reducing experimental SELEX cycles from 12–15 to 4–6 and accelerating discovery for BBB receptors [34,48]. Integration with molecular dynamics simulations and single-cell RNA-seq data will further enable patient-specific aptamer design tailored to disease-state BBB heterogeneity.

3. Theragnostic applications

Aptamers functionalized with fluorescent probes, allow real-time imaging of BBB crossing and therapeutic response, supporting precision medicine in neuro-oncology and neurodegenerative disorders [32]. Recent studies show potential for concurrent visualization and gene silencing in preclinical models [32].

Continued integration of advanced SELEX methodologies, machine learning-driven design, multifunctional nanoparticle hybrids, and theragnostic applications will unlock the full potential of aptamers. These advancements promise to transform the treatment landscape for neurological disorders, paving the way for safer, more precise, and personalized CNS therapies.

## Figures and Tables

**Table 1:** Comparison of aptamers with Traditional CNS Delivery Systems and antibodies for BBB crossing and Targeted Therapy.

Feature	Aptamers	Antibodies	Liposomes	Polymeric Nanoparticles	Viral Vectors (e.g., AAV)	References
Size (kDa)	~6–30 (20–100 nt)	~150	Variable (50–200 nm)	Variable (10–200 nm)	~20–30 (capsid)	[3,4,5,6,7,8]
BBB Penetration	High (via RMT; small size aids transcytosis)	Low (requires engineering, e.g., bispecific)	Limited (passive; needs targeting)	Inconsistent (often low without modification)	Moderate to high (natural tropism but variable)	[2,3,4,5,8,9,10,11]
Specificity/Affinity	High (nM–pM Kd)	Very high (pM Kd)	Low (unless functionalized)	low (surface modifiable)	High (tropism-dependent)	[3,4,5,6,8,9]
Immunogenicity	Low	High	Low	Low to moderate	High	[3,4,5,6,8,12]
Stability	Moderate to High (chemically modifiable)	Moderate (sensitive to environment)	Moderate	High	High	[3,4,5,7,8,13,14]
Production Cost	Low (chemical synthesis)	High (recombinant)	Moderate	Moderate	High	[3,4,5,6,8,12]
Toxicity Risks	Low [6,12]	Moderate (immune reaction)	Low	Low to moderate (accumulation)	High (inflammatory response)	[3,4,5,6,8,12]
Cargo Capacity	Limited (requires conjugation)	Moderate (conjugates)	High (encapsulation)	High	Limited (packaging size)	[3,4,5,8,11]
Key Advantages for CNS	Ease of modification, low cost, superior tissue penetration	Established clinical use	Biocompatible encapsulation	Versatile design	Efficient gene transfer	[3,4,5,6,8,10,12,14]
Key limitations	Nuclease sensitivity (mitigated by modifications)	Poor intrinsic BBB crossing	Lack of specificity	Inconsistent penetration/toxicity	Immunogenicity, mutagenesis risk	[3,4,5,8,12,13]

**Table 2:** Comparison of SELEX variants for aptamer selection, with focus on BBB-targeted applications.

SELEX Variant	Description	Strengths	Limitations	Examples in BBB/Aptamer Delivery Context	References
Classic SELEX	Uses purified proteins to isolate and identify specific binders through multiple rounds of binding, separation, and amplification.	Well-established; high affinity for known targets; controllable conditions.	Lacks physiological context; may not yield functional in vivo binders.	Foundational method for purified BBB receptors (e.g., TfR or LRP1 proteins) but limited brain penetration in vivo.	[22,23]
Cell-SELEX	Uses whole live cells (e.g., hBMECs) for selection against native surface molecules.	Targets native conformations; reduces off-target binding with counter-selection.	Limited to cell-surface targets; no full in vivo physiology (e.g., flow, clearance).	Selection against brain microvascular endothelial cells (e.g., hCMEC/D3 or bEND3 lines) for cross-species BBB-reactive aptamers; useful for endothelial receptor targeting but less efficient for functional transcytosis.	[24]
In vivo SELEX	Library injected into animals; bound aptamers recovered from target organ (e.g., brain after perfusion).	Accounts for full physiology (circulation, BBB interactions, clearance); yields true brain-penetrating aptamers.	Time-consuming (>15 rounds often needed); animal use/ethics; low recovery yields; species differences.	Identification of brain capillary binding and parenchymal-penetrating RNA aptamers (e.g., A15) in mice.	[25]
Microfluidic-based MPS-SELEX (Microphysiological System-SELEX)	Uses human BBB-on-a-chip (e.g., iPSC-derived BMECs with astrocytes/pericytes) under flow; permeable sequences collected and amplified.	Mimics human BBB physiology (shear stress, tight junctions); function-based selection for transcytosis; human-relevant.	Emerging technology; chip fabrication needed; fewer rounds but validation required.	Identification of hBS01 DNA aptamer enabling clathrin-mediated transcytosis and 10-fold enhanced cargo delivery across human BBB model.	[10]

**Table 3:** Comparison of DNA and RNA Aptamers for BBB delivery.

Property	DNA Aptamers	RNA Aptamers	References
Structure	Deoxyribonucleotides; stable secondary structures (e.g., stem-loops, G-quadruplexes)	Ribonucleotides; complex tertiary structures (e.g., helices, non-canonical motifs)	[27,28]
Stability	High thermal stability (>60 °C); resistant to nucleases (half-life >48 h without modifications)	Susceptible to RNases (half-life minutes); requires modifications (e.g., 2′-fluoro) for >48 h	[7,13,29]
Binding Affinity (K_d_)	low to mid-nanomolar (typically 10–100 nM for TfR-targeted)	Picomolar to low nanomolar (optimized examples down to ~20–30 pM for TfR-targeted)	[28,30]
Synthesis cost	Low	Higher due to modifications	[12]
BBB Applications	Stable for large constructs (e.g., plasmids, CRISPR); TfR targeting in AD/tauopathy models; delivering DNA aptamers as sensors across BBB revealing ATP decrease in AD models	Versatile for small cargos (e.g., siRNA, miRNA); LRP1/IR targeting in metabolic stress; Targeted delivery of RNA therapies	[9,31,32,33]
Advantages	Cost-effective, sustained delivery for chronic disorders	High versatility for dynamic targets	[18]
Challenges	Less structural complexity	Nuclease degradation	[14]

## Data Availability

No new data were created or analyzed in this study. Data sharing is not applicable to this article.

## Abbreviations

The following abbreviations are used in this manuscript:

BBB	blood–brain barrier
TfR	transferrin receptor
SELEX	Systematic Evolution of Ligands by Exponential Enrichment
ML	machine learning
MD	molecular dynamics
MPS	Microphysiological system
LRP-1	low-density lipoprotein receptor-related protein 1
GLUT1	Glucose Transporter 1
CNS	central nervous system
BDNF	brain-derived neurotrophic factor
LNP	lipid nanoparticles
RMT	receptor-mediated transcytosis
